# Exploration of the effect of PUM1/Cripto-1 pathway on ferroptosis by regulating macrophage polarization in allogeneic blood transfused mice

**DOI:** 10.18632/aging.204818

**Published:** 2023-06-29

**Authors:** Man-Di Wu, Yan Zhang, Huan Wang, Ke Yue, Yu Bai, Lai-Wei You, Ying-Hui Cui, Jian-Rong Guo

**Affiliations:** 1 School of Clinic Medicine, Ningxia Medical University, Ningxia 750004, China; 2 Postgraduate Training Base in Shanghai Gongli Hospital, Ningxia Medical University, Shanghai 200135, China; 3 School of Medicine, Shanghai University, Shanghai 200444, China; 4 Department of Anesthesiology, Shanghai Gongli Hospital, Naval Military Medical University, Shanghai 200135, China

**Keywords:** allogeneic blood transfusion, macrophage polarization, PUM1/Cripto-1 pathway

## Abstract

Background: To study the link between macrophage polarization, PUM1/Cripto-1 pathway and ferroptosis in the allogeneic blood transfusion setting.

Methods: This is an exploratory research. The purpose of this study was to investigate the effect of PUM1/Cripto-1 pathway on ferroptosis by regulating macrophage polarization in allogeneic blood transfused mice. Establish *in vitro* cell models and *in vivo* rat models. To find out whether PUM1 and Cripto-1 were expressed, RT-qPCR and Western blot analyses were employed. The macrophage polarization markers iNOS, TNF-, IL-1, IL-6, Arg-1, and IL-10 were utilized to identify M1 and M2 macrophages. JC-1 staining was used to detect ATP membrane potential in peripheral blood macrophages.

Results: In animal experiments, expression of Cripto-1 was negatively regulated by PUM1 and promoted M1 type polarization of macrophages. Allogeneic blood transfusion assured good state of macrophage mitochondria. Allogeneic blood transfusion inhibited ferroptosis in macrophages by affecting the PUM1/Cripto-1 pathway. In cell experiments, PUM1 regulated Cripto-1 in mouse macrophage RAW264.7. Polarization of RAW264.7 cells was regulated by the PUM1/Cripto-1 pathway. The effect of PUM1/Cripto-1 pathway on macrophage ferroptosis in cell experiments was consistent with that in animal experiments.

Conclusions: In this study, through *in vivo* cell experiments and *in vitro* animal experiments, it was successfully proved that PUM1/Cripto-1 pathway affected ferroptosis by regulating macrophage polarization in allogeneic blood transfused mice.

## INTRODUCTION

Monocytes, which are descended from bone marrow precursor cells, are the ancestors of macrophages, which are white blood cells that reside in tissues. Both macrophages and monocytes are phagocytic cells found in vertebrates, contributing to both general (innate immunity) and specialized (cellular immunity) defense. Their main function is to phagocytose (digest) cellular waste and pathogens that are present as fixed or free cells. They also stimulate lymphocytes and other immune cells to fight infections [1, 2]. Macrophages are immune cells that perform a wide range of tasks and are key research subjects in cell phagocytosis, cellular immunity, and molecular immunology [3].

As a flexible and pluripotent cell type, macrophages exhibit glaring functional changes both *in vitro* and *in vivo* under the impact of different microenvironments. Based on their activation states and roles, macrophages are divided into two types: M1 type (classically activated macrophages) and M2 type (alternatively activated macrophages). The two extremes of the spectrum of functional states that macrophages can be in are M1 and M2, respectively [4, 5]. By secreting pro-inflammatory cytokines and chemokines (like TNF-α, IL-1, and iNOS) and professionally presenting antigens, M1-type macrophages play an important role in immune regulation, tissue damage repair, and fibrosis; in contrast, M2-type macrophages have only weak antigen-presenting ability and down-regulate immune responses by secreting inhibitory cytokines like IL-10 or TGF-β [6]. Identification of macrophage types by phenotype is critical for researching macrophage functions under various physiological and pathological situations.

Some studies have shown that allogeneic blood transfusion causes macrophages to exhibit anti-inflammatory, that is, M2-type polarization [7]. We speculated that the effect of allogeneic blood transfusion on macrophage polarization was related to the regulation of PUM1/Cripto-1 pathway. No evidence has been found that Cripto-1 regulated macrophage polarization. However, the literature shows that overexpression of Cripto-1 can promote the expression of anti-inflammatory factor IL-10 and pro-inflammatory factors IL-1β, TNF-α, IL-6 in macrophages, and at the same time Cripto-1 also activates NF-κB pathway and increases the expression of IκB [8]. Considering the activating effect of Cripto-1 on the NF-κB pathway, it can be inferred that Cripto-1 should mainly promote M1 polarization [9]. Based on this, we reasoned that allogeneic blood transfusion should reduce the expression of Cripto-1, which was consistent with the two trends towards macrophage polarization.

There is a certain correlation between macrophage polarization and ferroptosis. Studies have shown that inhibition of the ferroptosis pathway in inferior arachnoid hemorrhage inhibits the activation of microglia and the release of inflammatory factors, suggesting that inhibition of the ferroptosis pathway may inhibit microglial M1 polarization [10]. In macrophages, however, studies have found that the autophagy-dependent ferroptosis pathway may activate M2 polarization in tumor-associated macrophages [11]. However, other studies have shown that ferroptosis can trigger the activation of pro-inflammatory pathways in tumor-associated macrophages, suggesting that it may promote M1 polarization [12]. All of these indicate that there is a correlation between macrophage polarization and ferroptosis, but its regulatory mechanism is complex and may have different trends in different environments. However, the activation or inhibition of the ferroptosis pathway also exists in macrophages itself, and its research is relatively less [13]. PUM1 can bind to Cripto-1, and it plays an anti-inflammatory role in physiological processes [14]. There is no report between PUM1 and ferroptosis. Based on the above, we hypothesized that M1 polarization of macrophages is associated with ferroptosis, and the promotion of M1 polarization may be related to the activation of the ferroptosis pathway.

The purpose of this study was to investigate the effect of PUM1/Cripto-1 pathway on ferroptosis by regulating macrophage polarization in allogeneic blood transfused mice. Establish *in vitro* cell models and *in vivo* rat models. To find out whether PUM1 and Cripto-1 were expressed, RT-qPCR and Western blot analyses were employed. The macrophage polarization markers iNOS, TNF-, IL-1, IL-6, Arg-1, and IL-10 were utilized to identify M1 and M2 macrophages. JC-1 staining was used to detect ATP membrane potential in peripheral blood macrophages. The activity of complexes I, II, III, IV were detected by ELISA. The total iron content, Fe^2+^ content and ROS, MDA and GSH content were detected by corresponding kits. The binding of PUM1 and Cripto-1 was detected in RAW264.7 cells by GST-PULL DOWN and Co-immunoprecipitation (CO-IP). Using flow cytometry, the M1 and M2 macrophage markers CD86 and CD206 were found. The expression of the proteins GPX4 and Nrf2, which are connected to ferroptosis, was discovered via Western blot analysis.

## Results and Discussion

### Animal experiments

### Expression of Cripto-1 was negatively regulated by PUM1 and promoted M1 type polarization of macrophages

**Figure 1:**
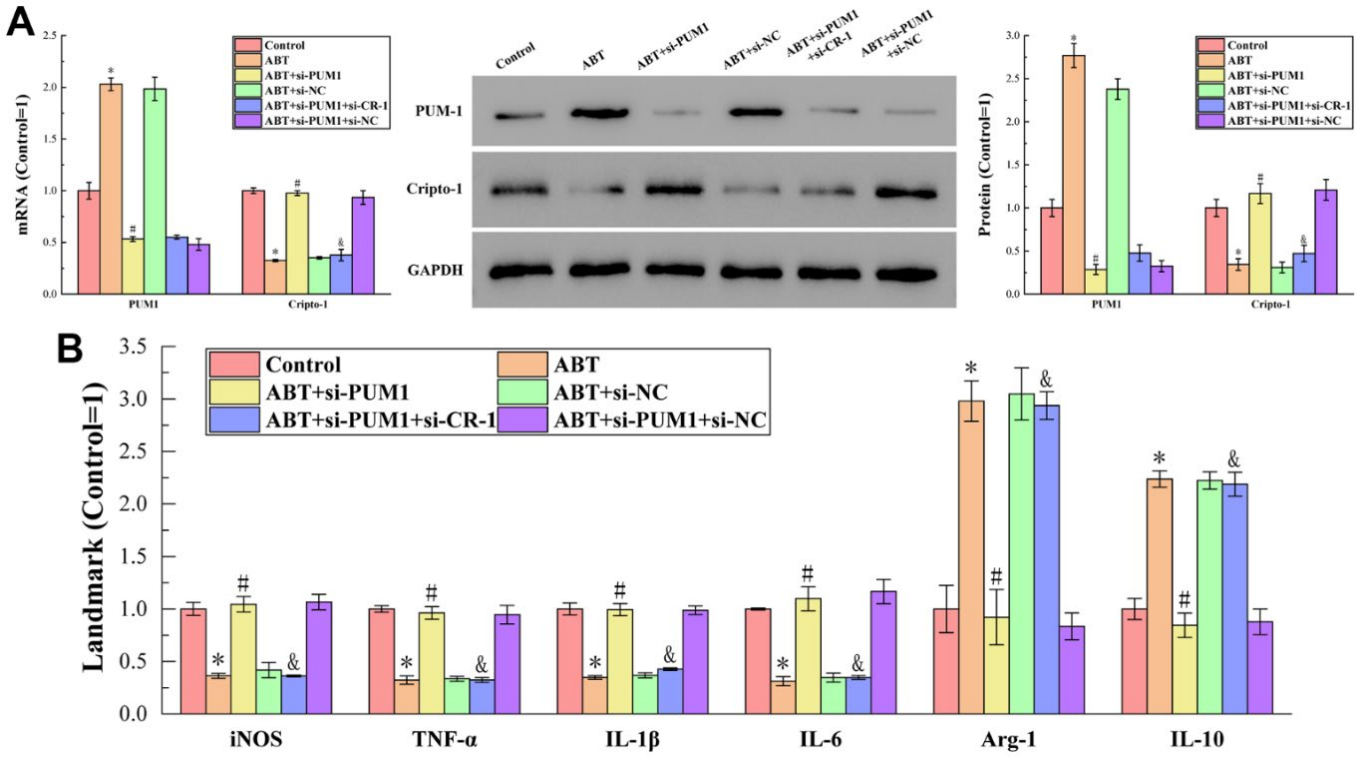
(**A**) The results of RT-qPCR and Western blot analysis. (**B**) The results of ELISA detection. The specific values were: 6.00 ± 0.38 U/L (iNOS), 938.94 ± 28.84 ng/L (TNF-α), 122.77 ± 7.12 ng/L (IL-1β), 123.13 ± 1.14 pg/mL (IL-6), 6.45 ± 1.45 U/L (Arg-1), 557.30 ± 55.87 pg/mL (IL-10). The symbol * means *p* < 0.05 compared to Control group. The symbol # means *p* < 0.05 compared to ABT group. The symbol and means *p* < 0.05 compared to ABT+si-PUM1 group.

Figure 1A displays the outcomes of RT-qPCR and Western blot analysis to find the amounts of PUM1 and Cripto-1 mRNA and protein expression in the blood of mice in each group. The figure showed that the PUM1 mRNA and protein expression levels in the ABT (allogeneic blood transfusion) group were higher than those in the control group. PUM1 gene expression was inhibited, and as a result, PUM1 mRNA and protein expression levels fell. When the gene expression of Cripto-1 was further inhibited on this basis, the mRNA and protein expressions of PUM1 were not affected, which indicated that Cripto-1 had no regulatory effect on PUM1. Furthermore, when the expression of PUM1 increased, the expression of Cripto-1 was inhibited, and when the expression of PUM1 decreased, the expression of Cripto-1 increased. It proved that the expression of PUM1 negatively regulated the expression of Cripto-1.

Figure 1B displays the findings of an additional ELISA detection that measured the expression levels of M1 macrophage markers (iNOS, TNF-, IL-1, IL-6) and M2 macrophage markers (Arg-1, IL-10) in the blood of mice in each group. As seen in the figure, when the expression of Cripto-1 decreased compared to the Control group, the expression of M1 macrophage markers decreased, and the M2 macrophage markers increased in the opposite direction; conversely, when the expression level increased, the M1 macrophage markers increased, and the M2 macrophage markers decreased. This suggested that the expression of Cripto-1 might encourage macrophage polarization to M1 type while suppressing M2 type polarization.

Combining the results of RT-qPCR, Western blot and ELISA, we could conclude that allogeneic blood transfusion could promote the expression of PUM1, which in turn inhibited the expression of Cripto-1, and finally promoted the polarization of macrophages towards the M1 type. The relationship between macrophage polarization and ferroptosis has been demonstrated by many research results, but the regulatory mechanism is not clear due to the complex relationship. Therefore, we would further discuss its relevance in the blood setting of allogeneic blood transfusion in the follow-up procedure.

### Allogeneic blood transfusion assured good state of macrophage mitochondria

**Figure 2:**
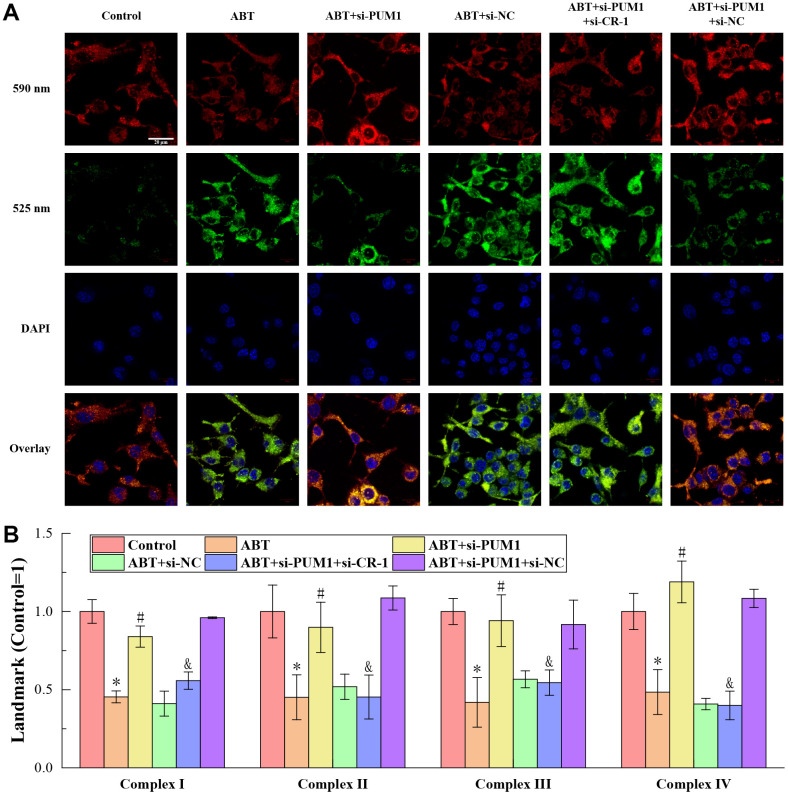
(**A**) The results of JC-1 staining. (**B**) The results of ELISA detection. The specific values were: 43.83 ± 3.31 U/mL (complexes I), 118.69 ± 20.04 U/mL (complexes II), 72.11 ± 6.01 U/mL (complexes III), 111.66 ± 12.90 U/mL (complexes IV). The symbol * means *p* < 0.05 compared to Control group. The symbol # means *p* < 0.05 compared to ABT group. The symbol and means *p* < 0.05 compared to ABT+si-PUM1 group.

The results of JC-1 staining to detect the membrane potential of macrophages in the blood of mice in each group are shown in Figure 2A. JC-1 is a cationic dye that can aggregate in mitochondria, mainly exists as monomer at low concentration, and emits mainly green light (~525nm); while at high concentration, it can form multimer, and the emission light is dominated by red light (~590 nm). The mitochondria itself has a certain polarity, the outer membrane is the negative electrode, and the inner membrane is the positive electrode. The potential difference is regulated by Ca^2+^, Na^+^ and H^+^ currents. When the mitochondria are in good condition, the uptake of JC-1 is small, so it mainly exists in the form of monomer in the mitochondria, and the ratio of green light intensity/red light intensity is high. When mitochondria are depolarized, the concentration of JC-1 in mitochondria is higher, mostly in the form of multimers, and the ratio of green light intensity/red light intensity decreases. The green light intensity/red light intensity of JC-1 staining of cells only depends on the membrane potential of mitochondria, and has nothing to do with the shape, volume and density of mitochondria, so it can better reflect the functional state of mitochondria [15]. As shown, in the Control, ABT+si-PUM1 and ABT+si-PUM1+si-NC groups, the red fluorescence intensity of JC-1 staining was higher and the green fluorescence intensity was lower, while the results in the other three groups were just the opposite. This showed that the state of mitochondria was negatively correlated with the expression of Cripto-1. When the expression of Cripto-1 was inhibited, the state of mitochondria was better, and vice versa.

Figure 2B shows the results of ELISA detection of mitochondrial respiratory chain complexes I-IV. The mitochondrial respiratory chain complexes are a series of protein complexes distributed on the inner membrane of mitochondria to transfer electrons and pump out protons, whose main function is to assist mitochondria to complete the ATP production process and provide energy for cells [16, 17]. As can be seen from the figure, the expression levels of complexes I-IV were consistent with the expression trend of Cripto-1 in each group. Allogeneic blood transfusion reduced the expression of complexes I-IV, maintained the rate of mitochondrial energy production at a normal level, and finally ensured the stability of macrophages.

### Allogeneic blood transfusion inhibited ferroptosis in macrophages by affecting the PUM1/Cripto-1 pathway

**Figure 3:**
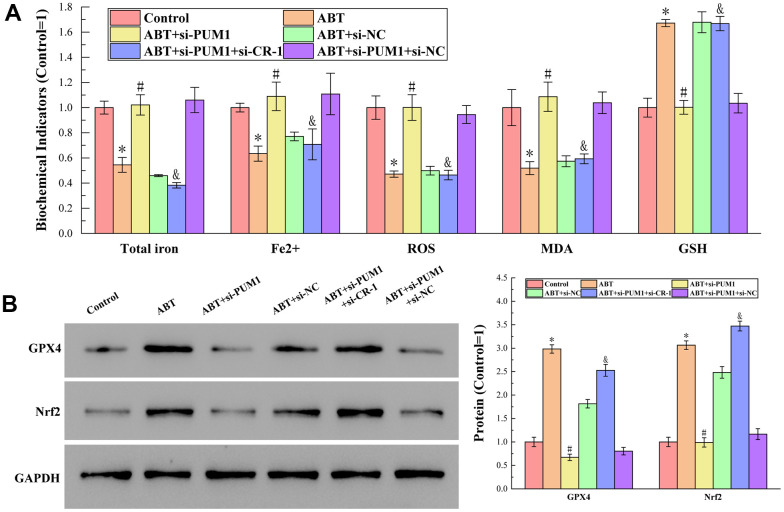
(**A**) The results of detecting total iron content, Fe^2+^ content, ROS, MDA and GSH content by corresponding test kits. The specific values were: 7.77 ± 0.40 nmol (Total iron), 3.18 ± 0.11 nmol (Fe^2+^), 2101.55 ± 194.88 RLU/mgprot (ROS), 12.24 ± 1.76 nmol/mL (MDA), 542.25 ± 40.64 μmol/L (GSH). (**B**) The results of Western blot analysis of protein GPX4 and Nrf2 content. The symbol * means *p* < 0.05 compared to Control group. The symbol # means *p* < 0.05 compared to ABT group. The symbol and means *p* < 0.05 compared to ABT+si-PUM1 group.

The results of detecting total iron content, Fe^2+^ content, ROS, MDA and GSH content by corresponding test kits are shown in Figure 3A. In the process of ferroptosis, Fe^2+^ is a cofactor of the reaction, and ROS and MDA are the products of the reaction. The presence of GSH enables activation of the enzyme GPX4 for liposomal peroxide reduction, thereby inhibiting ferroptosis [18–20]. It can be seen from the figure that compared with the Control group, the contents of total iron, Fe^2+^, ROS and MDA in the ABT group were decreased, while the content of GSH was increased. All data demonstrated that ferroptosis of macrophages was inhibited. Furthermore, the changes of each index in the experiment were positively or negatively correlated with the expression of Cripto-1, indicating that the ferroptosis of macrophages was regulated by Cripto-1 and the polarization of macrophages it affected.

The results of Western blot analysis of protein GPX4 and Nrf2 content are shown in Figure 3B. GPX4 inhibits ferroptosis by reducing liposomal peroxides by depleting GSH [21]. Nrf2 regulates many genes directly or indirectly involved in ferroptosis, thereby acting to inhibit ferroptosis [22]. It can be seen from the figure that compared with the Control group, the expressions of GPX4 and Nrf2 in the ABT group were increased, which further indicated that allogeneic blood transfusion could inhibit the ferroptosis of macrophages. At the same time, the expression levels of GPX4 and Nrf2 were also negatively correlated with the expression of Cripto-1, indicating that Cripto-1-regulated macrophage polarization was the key to affecting macrophage ferroptosis.

### Cell experiments

### PUM1 regulated Cripto-1 in mouse macrophage RAW264.7

**Figure 4:**
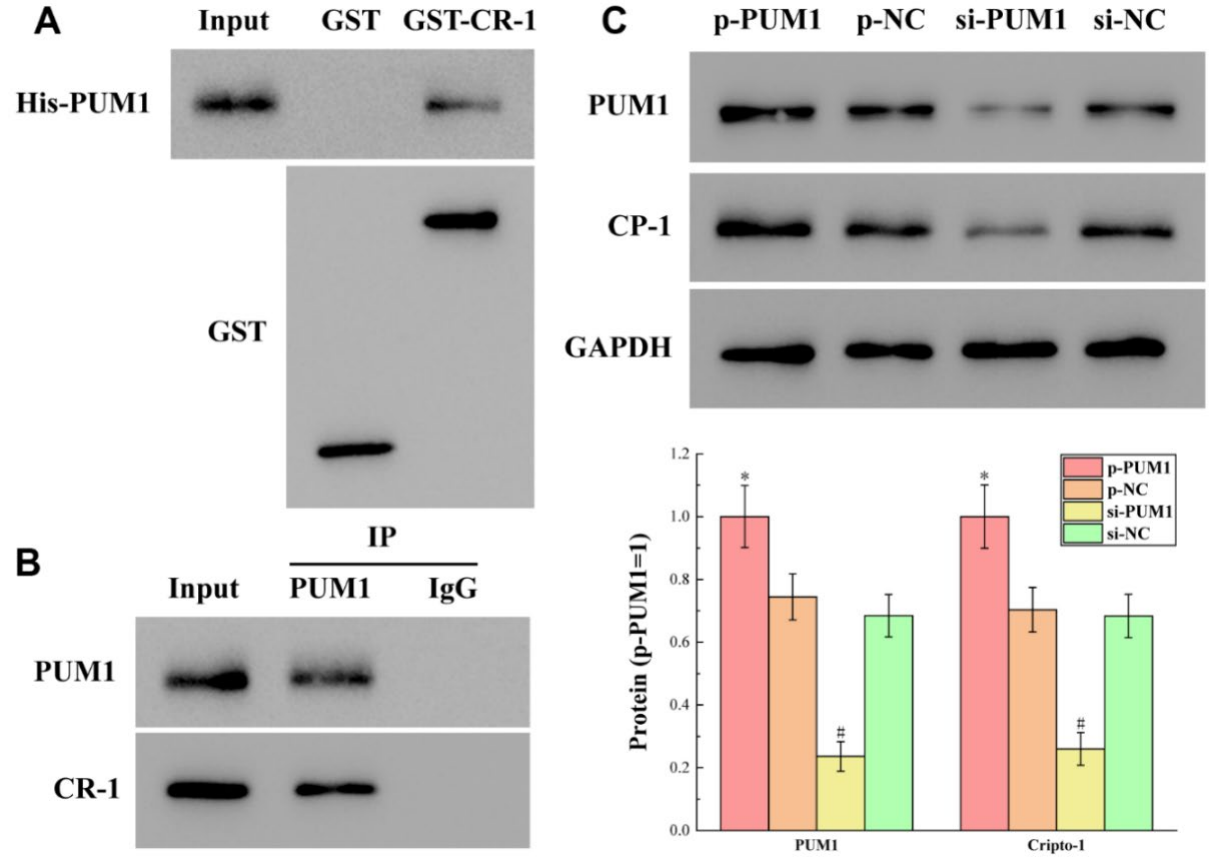
(**A**, **B**) The results of detecting the binding of PUM1 to Cripto-1 by GST pull-down and CO-IP experiments. (**C**) The results of the protein expression levels of PUM1 and Cripto-1 in each group in which the expression level of PUM1 was regulated by gene editing. The symbol * means *p* < 0.05 compared to p-NC group. The symbol # means *p* < 0.05 compared to si-NC group.

The results of detecting the binding of PUM1 to Cripto-1 by GST pull-down and CO-IP experiments are shown in Figure 4A, 4B. Figure 4A shows the presence of His-PUM1 bands in the GST-CR-1 (GST-Cripto-1) group. Figure 4B shows the presence of Cripto-1 bands in the PUM1 group. These two experiments jointly demonstrated that PUM1 was bound to Cripto-1, and that PUM1 was the regulator of Cripto-1.

At the same time, Figure 4C shows the protein expression levels of PUM1 and Cripto-1 in each group in which the expression level of PUM1 was regulated by gene editing. It can be seen from the figure that when the expression level of PUM1 increased, the expression level of Cripto-1 increased correspondingly, and vice versa. This showed that the regulation of Cripto-1 by PUM1 was a positive correlation regulation, that is, they rose and fell at the same time.

### Polarization of RAW264.7 cells was regulated by the PUM1/Cripto-1 pathway

**Figure 5:**
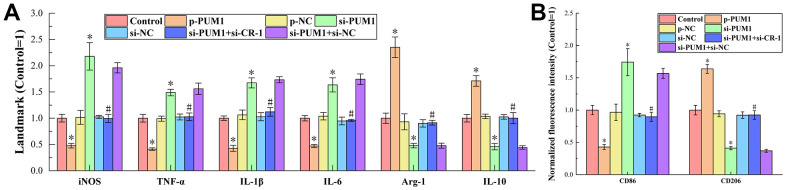
(**A**) The results of ELISA detection. The specific values were: 4.21 ± 0.31 U/L (iNOS), 769.18 ± 56.63 ng/L (TNF-α), 107.25 ± 4.66 ng/L (IL-1β), 100.43 ± 5.16 pg/mL (IL-6), 7.50 ± 0.73 U/L (Arg-1), 623.92 ± 44.92 pg/ mL (IL-10). (**B**) The results of flow cytometry detection. The symbol * means *p* < 0.05 compared to Control group. The symbol # means *p* < 0.05 compared to si-PUM1 group.

The findings of ELISA detection of macrophage M1- and M2-type polarization markers (iNOS, TNF-, IL-1, and IL-6) in each group are shown in Figure 5A. The picture shows that when PUM1 expression rose and Cripto-1 expression dropped relative to the Control group, the expression of M1-type polarization markers fell and the expression of M2-type polarization markers increased, indicating that macrophages were polarized towards the M2 type. The expression of M1-type polarization markers rose and the expression of M2-type polarization markers decreased when PUM1 expression was repressed and Cripto-1 expression was elevated, showing that macrophages were polarized in the M1-type direction. When the expression levels of PUM1 and Cripto-1 were both inhibited, the expression levels of M1-type polarization markers and M2-type polarization markers were comparable to those in the Control group, that is, macrophages did not polarize in a specific direction. Considering that PUM1 was used to regulate Cripto-1, Cripto-1 should be a direct factor in regulating the polarization direction of macrophages. The expression of M1-type polarization markers was positively correlated with the expression of Cripto-1, and the expression of M2-type polarization markers was negatively correlated with the expression of Cripto-1, that is, Cripto-1 promoted M1-type polarization of macrophages, suppressing its M2-type polarization.

Figure 5B shows the results of flow cytometry detection of the expression levels of M1 macrophage marker CD86 and M2 macrophage marker CD206 in each group. It can be seen from the figure that the expression trend of CD86 in each group was consistent with the trend of each M1-type polarization marker in ELISA detection, and the expression trend of CD206 in each group was consistent with the trend of each M2-type polarization marker. This again strongly supported the conclusion set out in the previous paragraph.

### The effect of PUM1/Cripto-1 pathway on macrophage ferroptosis in cell experiments was consistent with that in animal experiments

**Figure 6:**
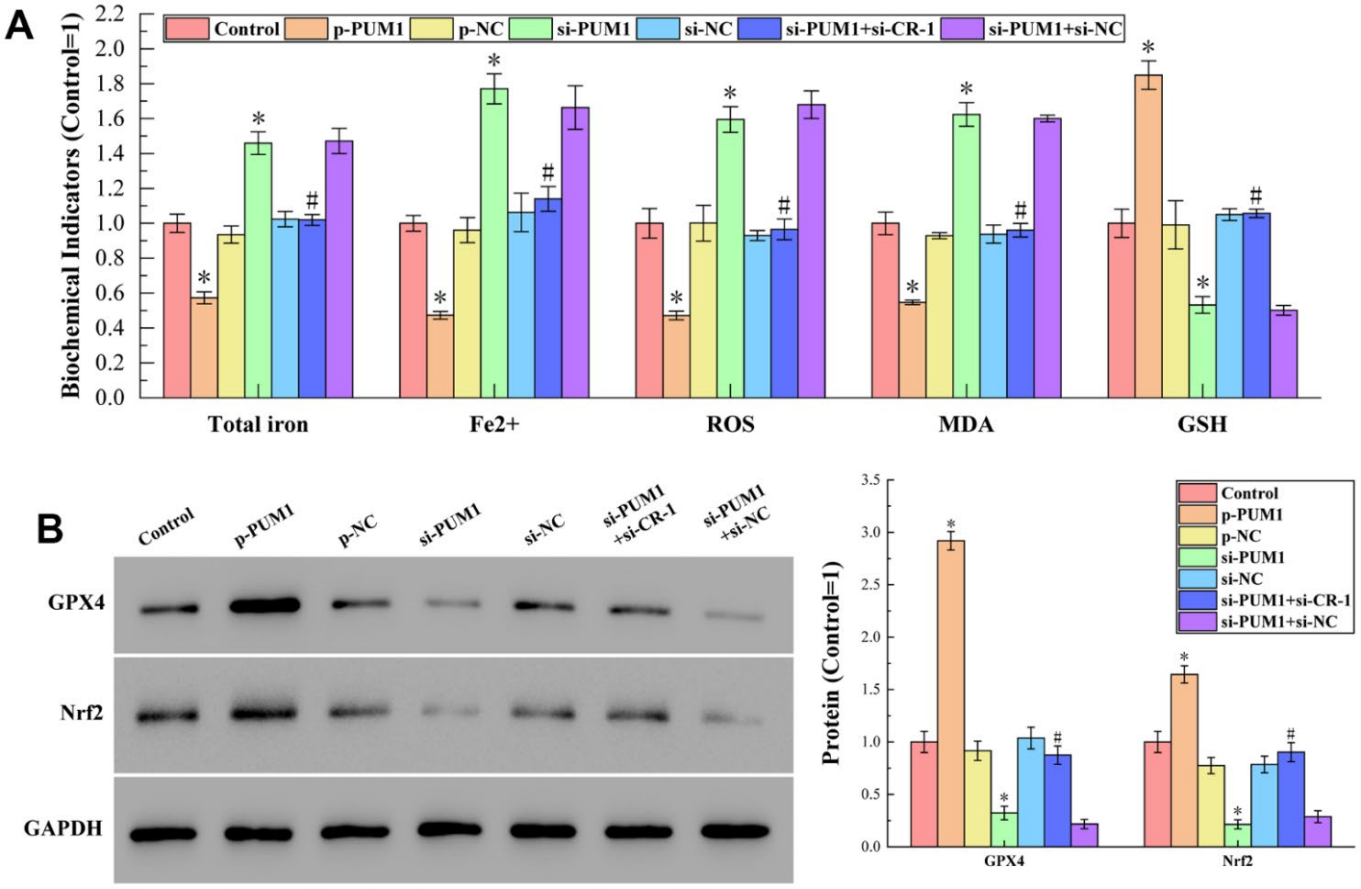
(**A**) The results of detecting total iron content, Fe^2+^ content, ROS, MDA and GSH content by corresponding test kits. The specific values were: 6.45 ± 0.35 nmol (Total iron), 3.40 ± 0.15 nmol (Fe^2+^), 2170.78 ± 183.53 RLU/mgprot (ROS), 15.09 ± 0.97 nmol/mL (MDA), 470.79 ± 38.25 μmol/L (GSH). (**B**) The results of Western blot analysis. The symbol * means *p* < 0.05 compared to Control group. The symbol # means *p* < 0.05 compared to si-PUM1 group.

The results of detecting total iron content, Fe^2+^ content, ROS, MDA and GSH content by corresponding test kits are shown in Figure 6A. Figure 6B shows the results of Western blot analysis of protein GPX4 and Nrf2 content. It can be seen from the figure that the affection of the PUM1/Cripto-1 pathway to macrophage ferroptosis by regulating the polarization of macrophages in the co-culture of human cord blood hematopoietic stem cells and RAW264.7 cells was completely consistent with the animal experiments on mice allogeneic blood transfusion. With consistent *in vivo* and *in vitro* experiments, we demonstrated that the PUM1/Cripto-1 pathway can affect ferroptosis by regulating macrophage polarization.

## CONCLUSIONS

In this study, through *in vivo* cell experiments and *in vitro* animal experiments, it was successfully proved that PUM1/Cripto-1 pathway affected ferroptosis by regulating macrophage polarization in allogeneic blood transfused mice. This provides a new conclusion for studying the complex relationship between macrophage polarization and ferroptosis, thus providing ideas for follow-up studies on new therapeutic and diagnostic options. Nevertheless, more in-depth studies are still needed to improve our conclusions and to better understand the effects of macrophage polarization on ferroptosis.

## MATERIALS AND METHODS

### Experiment design

### Animal experiments

This is an exploratory research. C57BL/6J mice were used as animal experimental models. Gene knockout or overexpression models were constructed by overexpression and knockout of PUM1 or Cripto-1 gene by tail vein injection in mice. The mice model was established by tail vein injection of allogeneic blood transfusion. The construction method was as follows: intubation of the femoral artery, removing the blood of 10% of the whole-body blood volume of the mice within 5-10 minutes, and then infusion of an equal volume of plasma, the infusion rate is less than or equal to 4 ml/h. RT-qPCR and Western blot analysis were used to detect the expression of PUM1 and Cripto-1 in mice blood. ELISA detection was used to detect M1 and M2 macrophage polarization markers iNOS, TNF-α, IL-1β, IL-6 (as M1 type markers) and Arg-1, IL-10 (as M2 type markers) in mice blood. JC-1 staining was used to detect ATP membrane potential in peripheral blood macrophages of mice in each group. The activity of complexes I, II, III, IV were detected spectrophotometrically. Using the appropriate kits, the total iron content, Fe^2+^ content, ROS (Reactive oxygen species), MDA (Malonaldehyde), and GSH (Glutathione) content were all determined. The expression of the ferroptosis-related proteins Nrf2 and GPX4 was determined by Western blot analysis (Nuclear factor erythroid 2-related factor 2).

### Cell experiments

The *in vitro* cell model was established by co-culture of human cord blood hematopoietic stem cells and mouse macrophage RAW264.7 cells. p-PUM1, si-PUM1, p-Cripto-1 and si-Cripto-1 were used to overexpress or inhibit the expression of PUM1 and Cripto-1, respectively. The source of human cord blood hematopoietic stem cells was Shanghai Gongli Hospital, Naval Military Medical University. RT-QPCR and Western blot analysis were used to detect the expression of PUM1 and Cripto-1 in each group. The binding of PUM1 and Cripto-1 was detected in RAW264.7 cells by GST-PULL DOWN and Co-immunoprecipitation (CO-IP). ELISA detection was used to detect M1 and M2 macrophage polarization markers iNOS, TNF-α, IL-1β, IL-6 (as M1 type markers) and Arg-1, IL-10 (as M2 type markers) in each group. M1 and M2 macrophage markers CD86 and CD206 were detected by flow cytometry (FCM). The total iron content, Fe^2+^ content and ROS, MDA and GSH content were detected by corresponding kits. Western blot analysis was used to detect the expression of ferroptosis-related proteins GPX4 and Nrf2.

### RT-qPCR analysis

To extract the total RNA from the cells, TRIzol reagent was applied to mouse blood cells or RAW264.7 cells. First strand cDNA was created using the Revert Aid TW first Strand cDNA Synthesis Kit. For the PCR analysis, the QuantiNova SyBr Green PCR Kit was utilized. Conditions for the reaction were pre-denaturation at 95° C for one minute, denaturation at 95° C for thirty seconds, annealing at 60° C for thirty seconds, and elongation at 72° C for thirty seconds.

### Western blot analysis

Collect cells from each group and fill each six-well plate with 200 μl of cell lysate. The cells were sonicated and then lysed for 1 hour on cold. At 4° C, the lysed cell sample was centrifuged for 15 minutes at 12,500 rpm. Then, in a clean centrifuge tube, transfer the supernatant from the centrifuge tube. Protein concentration was measured using a GAPDH protein measurement kit. The protein samples that were analyzed were kept at -80° C. The protein loading concentration in Western blot electrophoresis was 50 μg per well. After SDS-PAGE electrophoresis, the membrane was transferred and blocked. To utilize concentration, the proteins PUM1, Cripto-1, GPX4 and Nrf2 were diluted using a primary antibody (1: 500, anti-human, Thermo-Fisher, USA). The samples were shaken overnight at 4° C in an incubator. After washing with PBS, the samples were incubated for 30 minutes at room temperature in the dark with the secondary antibody (1: 1000, anti-human, Thermo-Fisher, USA). Finally, the developer was utilized for photography and development.

### ELISA analysis

On the ELISA-coated plate, five standard wells were placed. According to the specifications for concentration, the standard samples were put to the wells and serially diluted. 50 μl of sample were placed in each well. The test sample well and a blank control well were then placed. While the sample well underwent the identical procedures, the blank control well did not add sample or enzyme-labeled reagent. 10 μl of sample should be added after 40 μl of sample diluent has been added to the sample well. The sample has been diluted five times in total. After covering the plate with a sealing film, incubate it for 30 minutes at 37° C. Wash the plate with washing solution after incubation, then dry it. Once the wells have dried, add 50 μl of enzyme-labeled reagent to all but the blank well. Repeat the washing and incubation processes. Following the sequential addition of 50 μl of developer A and 50 μl of developer B to each well, color is developed at 37° C for 15 minutes in the dark. Next, 50 μl of stop solution is added to halt the reaction (the blue turns to yellow immediately). Measure the absorbance (OD value) of each well sequentially at 450 nm while setting the blank control well to zero.

### JC-1 staining

Peripheral blood macrophages were isolated and cells were cultured in six-well plates. Suspend 10^6^ cells with 1 ml of medium. The positive control well received 1 μl of the apoptosis-inducing substance (Carbonyl cyanide 3-chlorophenylhydrazone, CCCP, concentration of 50 mM) to produce its final concentration 50 μM. It should spend 5 minutes being incubated in a cell incubator. 10 μl of JC-1 (200 μM) should be added to each well, resulting in a final concentration of 2 μM. For 15 minutes, place it in a cell incubator. Cells were incubated, then centrifuged at 400 g for 3 min, discarding the supernatant. Add 2 ml of PBS to each well to resuspend the cells after two rounds of PBS washing, then repeat the centrifugation and supernatant removal procedure once. In order to see the cells under a fluorescent microscope, the cells were resuspended in 500 μl of PBS.

### Flow cytometry analysis

Each group’s cells were transferred to a 2 ml centrifuge tube, which was then spun at 1500 rpm for 5 minutes before the supernatant was removed. Use 0.1% Triton X-100 to fix at room temperature for 10 minutes after fixing with 4% paraformaldehyde (PFA) at 4° C for 30 minutes. After adding 200μl of the primary antibody diluted with PBA, incubate the mixture at 4° C for two hours, remove the supernatant by centrifuging, and then rinse with PBS. If more dilution is required, add 200 μl of PBA-diluted fluorescein-labeled secondary antibody, and incubate for 30 minutes at 4° C in the dark. The cells were then put back into a flow tube with 500 μl of PBS and identified using a flow cytometer.

### Quantification of total iron, Fe^2+^, ROS, MDA, GSH

In this experiment, the quantification kits for total iron content and Fe^2+^ content were determined by the Iron Assay kit from Abcam Company. Malondialdehyde (MDA), reduced glutathione (GSH) and reactive oxygen species (ROS) were quantified using the corresponding kits produced by Nanjing Jiancheng Bioengineering Institute (NJJCBIO). All experimental steps were carried out in strict accordance with the instructions in each kit, and the final statistical results were obtained.

### Statistical analysis

The student T test was the statistical technique employed in this assignment. The mean and standard deviation of the experimental data are presented. Software called SPSS 23.0 was used to do the statistical analysis. Software from Origin 2022b and Adobe Illustrator 2021 were used to create the figures.
